# Contraceptive methods and fertility testing in young adult survivors of childhood cancer

**DOI:** 10.1007/s10815-023-02908-7

**Published:** 2023-08-16

**Authors:** Brooke Cherven, Lauren F. Quast, James L. Klosky, Cynthia A. Gerhardt, Katja Baust, Gabriele Calaminus, Peter Kaatsch, Mariët Hagedoorn, Marrit A. Tuinman, Vicky Lehmann

**Affiliations:** 1 Aflac Cancer and Blood Disorders Center at Children’s Healthcare of Atlanta, Atlanta, GA USA; 2 Department of Pediatrics, Emory University School of Medicine, Atlanta, GA USA; 3 Center for Biobehavioral Health, Abigail Wexner Research Institute at Nationwide Children’s Hospital, Columbus, OH USA; 4 Departments of Pediatrics and Psychology, The Ohio State University, Columbus, OH USA; 5 Department of Pediatric Hematology and Oncology, University Hospital Bonn, Bonn, Germany; 6 German Childhood Cancer Registry (GCCR), Institute of Medical Biostatistics, Epidemiology and Informatics (IMBEI), University Medical Center Mainz, Mainz, Germany; 7 Department of Health Psychology, University of Groningen, University Medical Center Groningen, Groningen, The Netherlands; 8 Department of Medical Psychology, Amsterdam UMC Location University of Amsterdam, Meibergdreef 9, 1105AZ Amsterdam, The Netherlands; 9 Cancer Center Amsterdam (CCA), Amsterdam, The Netherlands

**Keywords:** Childhood cancer, Contraception, Young adult, Survivorship, Fertility testing, Oncofertility

## Abstract

**Purpose:**

Reproductive health is important, but often neglected in cancer survivorship care. This study explored contraceptive use and factors associated with fertility testing among young adult survivors of childhood cancer in Germany.

**Methods:**

Young adult survivors of childhood cancer were identified through the German Childhood Cancer Registry and completed a mailed survey. Survivors were queried regarding contraceptive use, reproductive goals, uncertainty about fertility, and completion or interest in fertility testing. Multivariable stepwise logistic regression models were used to calculate Odds Ratios (OR) and 95% confidence intervals (CI) as a means of identifying factors associated with completion of and interest in fertility testing.

**Results:**

Survivors (*N* = 472; 57.8% female; aged 23.3 ± 1.5 years, and 14.9 ± 5.0 years from diagnosis), reported high rates of contraceptive use, including 61.2% using a single method, 30.6% dual methods, and 8.1% no/less effective methods. Few survivors had completed fertility testing (13.0%), although 58.8% were interested. Having been diagnosed during adolescence (OR = 2.66, 95%CI: 1.39–5.09), greater uncertainty about fertility (OR = 1.16, 95%CI: 1.03–1.31), and use of dual contraceptive methods (OR = 1.94, 95%CI: 1.02–3.69) were associated with having completed fertility testing. Factors associated with interest in fertility testing included goals of wanting to have children (OR = 7.76, 95%CI: 3.01–20.04) and greater uncertainty about fertility (OR = 1.19 95%CI: 1.06–1.33).

**Conclusion:**

In this sample of young adults who survived childhood cancer, most reported contraceptive use. Few survivors had completed fertility testing, although more than half were interested. Interventions are needed to address potential barriers to fertility testing and help survivors manage fertility-related uncertainty.

## Introduction

Improved cancer treatments have led to increased survival rates for childhood cancer, which most commonly include leukemia, lymphoma, or central nervous system tumors; with current survival rates exceeding 80% in Germany [1], across Europe [2], and the United States [3]. However, many survivors are at risk for a variety of late effects that can adversely affect long-term functioning and quality of life [4]. For example, gonadotoxic therapies (e.g., radiation, alkylating chemotherapy) place survivors at increased risk for infertility [5–7]. Accordingly, most childhood cancer survivors treated with gonadotoxic therapies report reproductive concerns and worry about infertility [8–10]. These concerns often increase as survivors become older and more aware of the potential adverse effects of their prior disease and treatment. Once survivors enter their reproductive years and may consider future family building, uncertainty about their fertility may cause fear, worry, distress, guilt, anxiety, or sadness [11, 12]. In turn, this may also negatively affect dating and romantic relationships, due to fear of discussing possible infertility or disappointing their (potential) partner or partner’s family [12–14]. Notably, many survivors have inaccurate perceptions about their risk for treatment-related infertility [15–17] and their fertility status [18]. Fertility testing through semen analysis (for men) or hormonal evaluation (e.g., Anti-Mullerian Hormone [AMH]) together with antral follicle count through ultrasound (for women) may clarify such perceptions or mitigate uncertainty and distress by elucidating survivors’ fertility status and reproductive options. Previous studies have demonstrated an interest in fertility testing among cancer survivors, but few survivors report completing such testing [19–21]. Furthermore, factors influencing fertility testing remain understudied [20, 21].

While survivors of childhood cancer may experience challenges with fertility and family building, adolescence and young adulthood is also generally a time when people are at risk for *unplanned* pregnancies and sexually transmitted infections (STIs) due to risky sexual behavior and lack of effective contraceptive use [22]. Prevention of STIs is a priority among cancer survivors as they engage in risky sexual behaviors at rates similar to sibling controls [23, 24], but can have increased vulnerability for acquisition of and negative outcomes associated with STIs due to prolonged immunosuppression [25]. At the same time, survivors report lower rates of hormonal contraception use and higher use of emergency contraception (e.g., the morning-after pill) compared to the general population [26–28]. Contraceptive use has been associated with survivors’ beliefs about their fertility status, even after controlling for confirmed infertility [29]. Specifically, survivors who *believed* they were infertile were four times less likely to use contraception than survivors who believed they were fertile. This is concerning as survivors can have inaccurate perceptions of their true infertility risk [16, 18, 30] and remain vulnerable to STIs regardless of fertility status. The current study aims to describe contraceptive use in young adult survivors of childhood cancer, to explore the association between contraceptive use and fertility testing, and to identify sociodemographic and clinical factors associated with fertility testing.

## Methods

### Procedures

This study is part of the larger E-Surv collaboration which included two surveys: *VIVE* (PI: Calaminus) that considered medical late effects of childhood cancer treatment and *InRel* (PI: Lehmann) that focused on intimate relationships [31].

The German Childhood Cancer Registry (GCCR) randomly selected *N* = 2000 survivors of childhood cancer (see eligibility below). Survivors were invited to participate in both studies in a counterbalanced manner (i.e., 50% were invited to participate in VIVE first and InRel second, and vice versa). Information packets were mailed to survivors, and in case of non-response, a reminder was sent after six weeks. After three months, survivors were invited to participate in the second survey of this project (i.e., either InRel or VIVE) irrespective of their previous (non-) response unless they explicitly opted out.

Participants provided informed consent prior to completing the survey, and all procedures were described previously [31]. This study approved by the Medical Ethical Committee (#138/17) and data protection officer of the University Medical Center Bonn, Germany and developed in accordance with the Declaration of Helsinki.

### Eligibility

Eligible survivors were diagnosed with any type of cancer before age 18, were long-term survivors (≥ 5 years post-diagnosis), were emerging/young adults (i.e., aged 20–25 years), were registered at the GCCR, and living in Germany at the time of data collection (2018–2019). Due to logistical delays after eligible survivors had been identified, participants were aged 21–26 years at study participation.

Of the 2000 identified survivors, 526 completed the InRel survey. These completers were somewhat younger (23.3 vs. 23.9 years, *p* < 0.001) and more often female (34.9% of eligible females vs. 19.3% of eligible males, *p* < 0.001) than non-completers, but they did not differ by type of or age at diagnosis. Throughout the survey, participants were able to skip questions. For the current analyses, we excluded survivors who had children already (*n* = 29), who had not been pregnant/sired a pregnancy previously (*n* = 7), or who *un*successfully tried to become pregnant for more than one year (*n* = 6). Another *n* = 12 survivors missed data for these items, resulting in a final sample of *N* = 472.

### Measures

**Sociodemographic data** including sex, relationship status (single/partnered), and completed level of school education (low, middle, high) were self-reported by survivors. Age and clinical data (i.e., age at diagnosis, type of diagnosis, relapse) were supplied by the GCCR (Table 1). Age at diagnosis ranged between 0–17 years, and was used as such, as well as dichotomized into age at diagnosis during childhood (≤ 12 years) versus adolescence (13–17 years of age). Cancer diagnosis was categorized as leukemia, lymphoma, central nervous system (CNS)-tumors, and other/solid tumors.

**Table 1:** Sample characteristics of all participants

	Entire Sample	Sexually active (*n* = 369)		
	*N* = *472*	Single Contraception (*n* = *226)*	Dual Contraception (*n* = *113)*	No/less effective contraception (*n* = 30)
Characteristic	M ± SD, range	M ± SD, range	M ± SD, range	M ± SD, range
Age (years)	23.3 ± 1.5, 21–26	23.4 ± 1.4, 21–26	23.5 ± 1.5, 21–26	23.3 ± 1.6, 21–26
Age at diagnosis (years)	7.9 ± 4.8, 0–17	8.0 ± 5.1, 0–17	7.9 ± 5.0, 0–17	8.5 ± 4.7, 0–16
Years since diagnosis	14.9 ± 5.0, 6–26	14.8 ± 5.4, 6–26	15.0 ± 5.2, 6–25	14.3 ± 4.5, 6–22
Gender	*n* (%)	*n* (%)	*n* (%)	*n* (%)
Female	273 (57.8%)	139 (61.5%)	68 (60.2%)	18 (60.0%)
Relationship Status^a^				
Single	245 (52.1%)	81 (36.0%)	56 (49.6%)	15 (50.0%)
Partnered/married	225 (47.9%)	144 (64.0%)	57 (50.4%)	15 (50.0%)
Completed School Education^b^				
Low	36 (7.7%)	13 (5.8%)	8 (7.1%)	5 (16.7%)
Middle	90 (19.4%)	37 (16.4%)	23 (20.4%)	8 (26.7%)
High	339 (72.9%)	174 (77.0%)	80 (70.8%)	17 (56.7%)
Diagnosis				
Leukemia	179 (37.9%)	87 (38.5%)	46 (40.7%)	11 (36.7%)
Lymphoma	91 (19.3%)	52 (23.0%)	18 (15.9%)	5 (16.7%)
CNS tumor	102 (21.6%)	38 (16.8%)	19 (16.8%)	6 (20.0%)
Solid tumor/Other	100 (21.2%)	49 (21.7%)	30 (26.5%)	8 (26.7%)
Age at Diagnosis				
Childhood (≤ 12)	363 (76.9%)	163 (72.1%)	84 (74.3%)	22 (73.3%)
Adolescence (13 +)	109 (23.1%)	63 (27.9%)	29 (25.7%)	8 (26.7%)
Relapse				
No relapse	406 (86.0%)	202 (89.4%)	100 (88.5%)	26 (86.7%)
Relapse/SMN	66 (14.0%)	22 (10.6%)	9 (11.5%)	4 (13.3%)
Fertility Uncertainty				
Composite	6.4 ± 2.6, 3–13	6.5 ± 2.5, 3–13	6.3 ± 2.7, 3–13	6.7 ±2.5, 3–13
Goal to have biological child(ren)?^c^				
Yes	400 (85.5%)	199 (88.1%)	102 (90.3%)	23 (76.7%)
No	68 (14.4%)	25 (11.1%)	11 (9.7%)	7 (23.3%)
Completed Fertility Testing^a^				
Yes	61 (13.0%)	28 (12.4%)	23 (20.4%)	5 (16.7%)
No	409 (87.0%)	198 (87.6%)	89 (78.8%)	25 (83.3%)
Interest Fertility Testing^d^	408/409	198/198	88/89	24/25
Yes	240 (58.8%)	128 (64.6%)	50 (56.8%)	17 (70.8%)
No/Maybe	168 (41.2%)	70 (35.4%)	38 (43.2%)	7 (29.2%)

*SMN* subsequent malignancy, *M* mean, *SD* standard deviation

^a^*N* = 470, ^b^*N* = 465, ^c^*N* = 468; ^d^those without formal fertility testing

Education was categorized as Low = none/ ≤ 9 years basic school education, Middle = 10 years secondary education, High = university entrance qualification

A series of face-valid questions assessed contraceptive use, reproductive goals, uncertainty about fertility, and completion or interest in fertility testing:

**Contraceptive use** was assessed among survivors with previous sexual experiences. They were asked which method(s) they used to prevent pregnancy at last sexual intercourse, with responses being categorized as *hormonal methods* (birth control pills, injections, implants, the Nuva Ring, or [hormonal] intrauterine devices [IUD]), *barrier method* (condom), or *no/less effective* methods (none, withdrawal, rhythm/fertility awareness methods). One participant indicated use of a copper IUD and was included in the hormonal contraception group, due to its effectiveness in preventing pregnancies. These contraceptive methods were further categorized as single contraception (hormonal *or* barrier) or dual contraception methods (hormonal *and* barrier) to distinguish in their ability in preventing both pregnancies and STIs. Participants were also able to select that they were not using contraception because they were currently attempting a pregnancy.

**Reproductive goals** were assessed by asking participants if it is a goal in their life to have biological children. Responses were dichotomized as *no* versus *yes* (incl. currently trying, soon, not yet).

**Uncertainty** of fertility status was measured with three face-valid items, including whether survivors tend to wonder about, are unsure, and believe that they might be infertile. Answers to these 3 items were measured on 4 or 5-point scales (*never/not at all*—*always/to a great degree*) and summed to an uncertainty score (potential range: 3–13), with higher scores indicating greater uncertainty. Cronbach’s alpha was 0.75 in this sample.

Participants were asked if they had ever undergone **fertility testing** and thus, if they knew their fertility status (“*Have you have been tested to find out what your fertility status is?*”). Response options included: (a) never having been tested or having been tested with results indicating (b) infertility, (c) impaired/sub-fertility, (d) fertility, or (e) unclear results. For analyses, participants were categorized as having completed fertility testing versus not.

Participants *without* prior fertility testing were queried regarding their **interest** in fertility testing. This question also explained that fertility testing would include producing a semen sample for males or blood work and ultrasounds for females. Responses were categorized as interested (yes, would consider if offered) or not interested (uncertain or would not consider if offered). Participants were also asked about reason(s) if they were not interested in completing fertility testing.

### Statistical analyses

Descriptive statistics of all measures of interest are presented. Differences in contraceptive use by sociodemographic (sex, age, relationship status, education) and medical factors (type of, age at, and time since diagnosis) were tested using *χ*^*2*^, *t*-tests or ANOVAs, depending on the variable type and whenever subgroups were sufficiently large.

Multivariable stepwise logistic regression models were used to calculate Odds Ratios (OR) of whether survivors had completed fertility testing. All usable basic sociodemographic and medical factors (sex, age at study, relationship status, age at diagnosis (childhood vs. adolescence) and type of diagnosis (CNS vs. others) were entered in block 1 using *backward* selection to explore which factors play a significant role, followed by entering reproductive goals and uncertainty in block 2, and finally contraceptive use in block 3. The same analyses were conducted for interest in fertility testing (i.e., for survivors without prior testing). Post-hoc power analyses indicated ample power (> 0.9) for the above analyses to detect even small effects (*r* = 0.2) given a sample of *n* = 472 survivors.

## Results

### Sample characteristics

Participants (*N* = 472) were on average 23.3 years old (standard deviation [*SD*] = 1.5, range: 21.0–26.0 years) and 14.9 years from cancer diagnosis (*SD* = 5.0, range: 6.0–26.0 years). Most participants were female (57.8%), single (52.1%), and had completed a university entrance qualification (72.9%; Table 1). At diagnosis, participants were on average 7.9 years of age (*SD* = 4.8, range: 0.0–17.0 years), and most often diagnosed with leukemia (37.9%). Most participants had not experienced a relapse/second malignancy (86.0%; Table 1). Note that due to unequal subgroup sizes, school education and relapse were not considered in subsequent analyses.

### Contraception, reproductive goals, and uncertainty

Of all *N* = 472 participants, *n* = 92 reported never having had sex (and were not asked questions about contraception), *n* = 3 were currently trying to get pregnant, and *n* = 8 were missing. Thus, of all survivors (*n* = 369) with data on contraceptive use at last intercourse, *n* = 113 (30.6%) reported using dual methods (i.e., hormonal *and* barrier methods) to prevent pregnancy, *n* = 226 (61.2%) used a single method (*either* hormonal: *n* = 133; or barrier: *n* = 93), and *n* = 30 (8.1%) used no/less effective methods (*n* = 10 withdrawal, *n* = 13 no method, *n* = 6 rhythm, *n* = 1 did not recall). These 30 survivors who used no/less effective methods were not included in further analyses due to small subgroup size. These survivors did not appear different from all other survivors, although relatively more had only completed a basic school education (Table 1). However, differences could not be formally tested due to the small subgroup size.

When comparing survivors by single vs. dual methods of contraceptive use, there were no differences related to age, age at diagnosis, or time since diagnosis (*t*’s < 0.62, *p*’s > 0.53) nor by sex or type of diagnosis (*χ*^*2*^ > 2.69, *p* > 0.44). Yet, single survivors were more likely to use dual contraceptive methods (*n* = 56/137, 40.9%) than partnered survivors (*n* = 57/201, 28.4%; *χ*^*2*^ = 5.74, *p* = 0.017). In other words, most survivors who used single contraceptive methods were partnered (*n* = 144/226; 64.0%, Table 1). Multivariable models were not tested for the contraception methods because of the lack of difference in considered variables.

Most participants (*n* = 400, 85.5%) reported that having a biological child is a goal in their life. Uncertainty regarding fertility status varied (*M* = 6.4, *SD* = 2.6; range: 3–13), with higher scores indicating greater uncertainty, wonder, or worry about fertility status.

### Fertility testing

Most participants (87.0%; *n* = 409/470; *n* = 2 missing) had never been tested and did not know their fertility status. The most common reasons for not having completed fertility testing were that participants did not think of it (54.3%, *n* = 222), believed they were not at risk for infertility (21.8%, *n* = 89), planned to be tested when they were older (16.4%, *n* = 67) or in a relationship (7.3%, *n* = 30), and/or lacked knowledge about where/how to access testing (7.6%, *n* = 31; note that participants could indicate various reasons). Of the 61 participants who received fertility testing, 52.4% (*n* = 32) were told that they were fertile, 31.1% (*n* = 19) had impaired fertility, 13.1% (*n* = 8) reported being infertile, and 3.3% (*n* = 2) reported unclear test results.

**Fig. 1:**
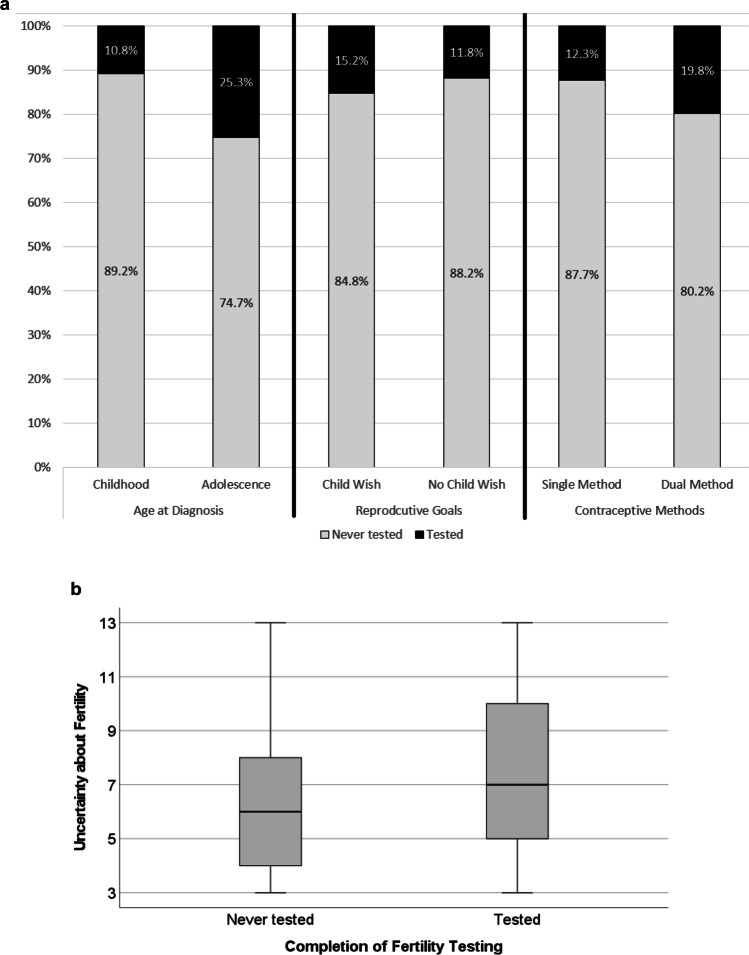
Significant factors in the final regression model by whether survivors had completed fertility testing or not (*n* = 331), showing (**a**) proportions of survivors stratified by age at diagnosis, reproductive goals, and contraceptive methods; and (**b**) average uncertainty scores

In a multivariable stepwise logistic regression model, survivors who had been diagnosed with cancer during adolescence as compared to childhood were almost 2.7 times more likely to report having completed fertility testing (OR = 2.66, 95%CI: 1.39–5.09, *p* = 0.003). Moreover, those with higher uncertainty about their fertility status (OR = 1.16, 95%CI: 1.03–1.31, *p* = 0.012), and those using dual (i.e., hormonal and barrier) contraceptive methods relative to using either/single methods (OR = 1.94, 95%CI: 1.02–3.69, *p* = 0.042, Table 2) were more likely to report having completed fertility testing (see Fig. 1a and b).

**Table 2:** Factors associated with completed fertility testing among cancer survivors (*n* = 331)

Variable	OR	95% CI	*p*
Age at Diagnosis*	2.66	1.39 – 5.09	**.003**
Reproductive Goals**	1.32	0.42 – 4.12	.638
Uncertainty	1.16	1.03 – 1.31	**.012**
Contraceptive Use***	1.94	1.02 – 3.69	**.042**

*CI* confidence interval, *OR* odds ratio; bolded *p*-values indicate significance at *p* < .05

^*^dichotomized as childhood (0) vs. adolescence (1)

^**^dichotomized as never/maybe (0) vs. yes (1)

^***^dichotomized as single (hormonal *or* barrier) method (0) vs. dual (hormonal *and* barriers) methods (1)

Variables in the model: block 1 [backward selection]: sex, age, age at diagnosis (childhood vs. adolescence), diagnosis (CNS vs. others), relationship status (partnered vs. single), block 2 [enter]: reproductive goals, uncertainty, block 3 [enter]: contraceptive use (single vs. dual methods)

### Interest in fertility testing

**Fig. 2:**
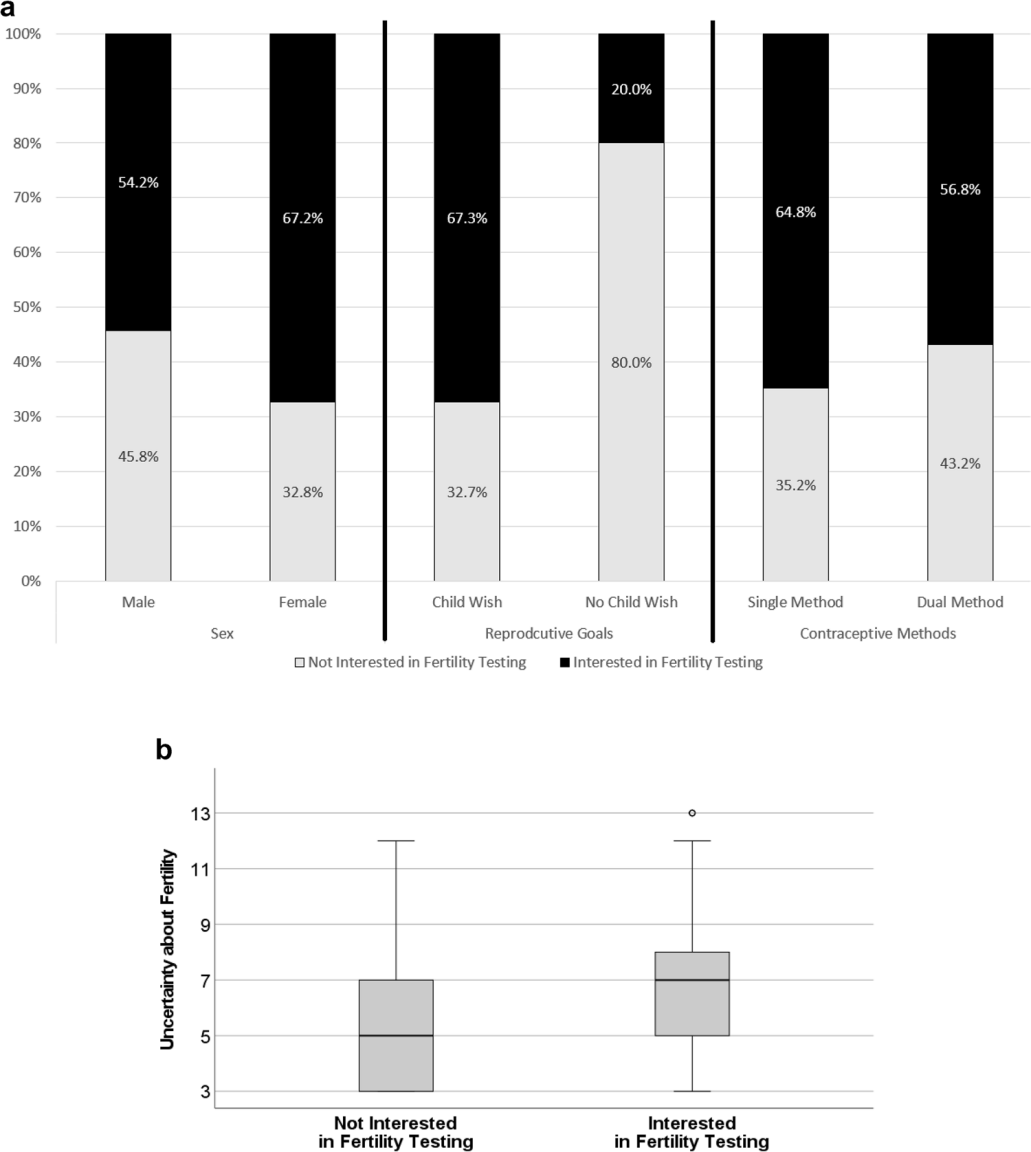
Significant factors in the final regression model by whether survivors were interested in fertility testing or not (*n* = 281), showing (**a**) proportions of survivors stratified by sex, reproductive goals, and contraceptive methods; and (**b**) average uncertainty scores

Of those *n* = 409 survivors without formal fertility testing, *n* = 408 provided responses about whether they would obtain fertility testing if it was offered to them. Of these, 58.8% (*n* = 240/408) participants responded yes, while 41.2% (*n* = 168/408) would decline testing (never: *n* = 18, 4.4%; undecided: *n* = 150, 36.8%). Factors associated with interest in obtaining fertility testing included wanting to have children in the future (OR = 7.76, 95%CI: 3.01–20.04, *p* < 0.001) and increased uncertainty about fertility (OR = 1.19, 95%CI: 1.06–1.33, *p* = 0.003; Table 3; Fig. 2a and b). Participants who indicated they would likely decline testing (*n* = 168) also provided reasons, including that they did not think they were at risk (40.5%, *n* = 68/168), did not want to have kids (15.5%, *n* = 26/168), or would be uncomfortable with test procedures (14.3%, *n* = 24/168). A smaller portion of participants also reported concerns regarding cost of procedures, feeling embarrassed, or fearing the results of fertility testing.

**Table 3:** Factors associated with interest in fertility testing among cancer survivors (*n* = 281)

Variable	OR	95% CI	*p*
Sex	1.51	0.89—2.58	.127
Reproductive Goals*	7.76	3.01—20.04	** < .001**
Uncertainty	1.19	1.06—1.33	**.003**
Contraceptive Use**	0.69	0.40—1.20	.184

*CI* confidence interval, *OR* odds ratio; bolded *p*-values indicate significance at p < .05

^*^dichotomized as never/maybe (0) vs. yes (1)

^**^dichotomized as single (hormonal *or* barrier) method (0) vs. dual (hormonal *and* barrier) methods (1)

Variables in the model: block 1 [backward selection]: sex, age, age at diagnosis (childhood vs. adolescence), diagnosis (CNS vs. others), relationship status (partnered vs. single), block 2 [enter]: reproductive goals, uncertainty, block 3 [enter]: contraceptive use (single vs. dual methods)

## Discussion

In this study of young adult survivors of childhood cancer, rates of contraceptive use were high, with almost one-third of survivors using both hormonal and barrier contraception. A small proportion of survivors had completed fertility testing, although over half of never-tested survivors reported interest in fertility testing. Uncertainty about fertility was identified as relevant for both having been tested, as well as being interested in testing. A cancer diagnosis during adolescence and using dual contraceptive methods were also associated with having completed testing, while goals of having a child in the future were strongly associated with interest in fertility testing.

Most (> 90%) participants reported using one or more forms of contraception during their last intercourse, which is similar to reports of German national studies where < 10% of healthy young adults report using *no* contraception at last intercourse, and almost one-third report using both hormonal and barrier contraception methods [32, 33]. Of note, hormonal contraception (e.g., the pill), is accessible through standard gynecological care and covered by health insurance for youth in Germany under the age of 18 years. For young adults 18–22 years, there is a minimal co-pay, and after the age of 22, individuals must pay in full (typically 10–20€ per month). Youth in Germany is typically covered under their parents’ health insurance until age 25, unless they have an own income. Additional forms of contraception (e.g., condoms) are also relatively inexpensive in Germany. In other cancer survivor populations, rates of contraceptive use were lower (47–84%) [34] and survivors were less likely than healthy peers to use contraception, but underlying reasons remain unclear [26]. In a sample of young adult female cancer survivors in the United States, contraceptive use was similar (84%) to our findings, but hormonal contraceptive methods were used by fewer participants (< 50%), likely due to the contraindication for hormonal methods among breast cancer survivors [29]. We also demonstrated that a larger proportion of single than partnered survivors reported dual contraceptive methods. The cancer survivor population is vulnerable to infection due to prolonged immunosuppression, making prevention of STIs a priority. Sexual health counseling for young adult cancer survivors should include discussing the specifics of hormonal methods protecting against pregnancy only and barrier methods against STIs and pregnancy. Despite high efficacy rates, IUDs were reported by a small proportion in this study, as well as young adults from the general German population [32].

Similar to previous studies of young adult cancer survivors, our findings demonstrate that far more survivors are interested in testing, than those who actually completed fertility testing [20, 21]. The most common reason for not having completed fertility testing was that survivors ‘did not think of it.’ This may partially be due to their still rather young age (≤ 26 years) and not yet desiring to have children. However, considering the level of interest in testing, clinical programs should raise awareness of fertility testing options and incorporate counseling and referrals for interested survivors [35, 36]. The cost of fertility testing may also be a barrier for some young adult cancer survivors, which varies between countries, and should be discussed during fertility-related counseling.

Dual (relative to single) contraceptive use was associated with a greater likelihood of having completed fertility testing. Among those who completed fertility testing, half were told they were fertile, likely encouraging them to prevent unintended pregnancies and resulting in using more than one contraceptive method. It may also be that survivors who had undergone fertility testing are more engaged in reproductive healthcare, having received additional counseling regarding pregnancy versus STI prevention, leading to dual methods of contraception.

Greater uncertainty regarding fertility was associated with interest in fertility testing, which aligns with prior research [21, 37]. However and somewhat surprisingly, greater uncertainty was also associated with completion of fertility testing. While fertility testing may elucidate fertility potential for some survivors, for others, results may be inconclusive, resulting in increased uncertainty. In our sample, one-third of survivors who had completed testing reported their fertility as ‘impaired’ or ‘unclear,’ which likely contributes to continued or maybe even heightened uncertainty. Moreover, female survivors who received testing and were told they were currently fertile, likely also received counseling regarding the decline of their fertility with age, and the potential for a shortened reproductive window due to gonadotoxic treatments. This may result in perceived pressure to have children earlier [12, 38], while still creating uncertainty about whether or not they will be able to have children in time. These are important topics to revisit over time and repeated fertility testing may be necessary to clarify possible declines in fertility with age. In contrast, male survivors, may experience reduced uncertainty following semen analysis, which provides typically more conclusive results. However, men are also often advised that even if infertile, their semen production may spontaneously recover –potentially contributing to some feelings of uncertainty. Regardless of sex, fertility testing is not a definitive prediction of future fertility, as many factors can contribute to infertility, including partners’ health and other lifestyle/behavioral factors. As uncertain fertility status can be associated with distress, survivors may benefit from counseling/psychological support [12].

Most participants (85.5%) reported a desire for having biological children, an important developmental milestone for many young adults and most childhood cancer survivors [19, 39]. In the current study, desire for future children was strongly related to survivors’ interest in fertility testing, but not related to having completed testing. This may be due to lack of awareness regarding testing options or how to schedule and complete testing despite interest, or survivors delaying assessment until they are older or ready to attempt pregnancy. Cost of fertility testing can vary, depending on different tests required for men and women, healthcare infrastructure and insurance coverage, which may also be a barrier to accessing fertility-related services. It is important that clinicians support survivors in achieving developmental goals by discussing fertility testing and related options, particularly when survivors have expressed interest in having children. Infertility and uncertain fertility status have been linked to poor mental health outcomes for childhood cancer survivors, including psychological burden and negative effects on romantic relationships, further highlighting the importance of providing survivors with ongoing information and support [10]. As efforts are underway to expand and standardize survivorship care in Germany [40], education and counseling regarding fertility testing should be incorporated into standard survivorship care.

Survivors diagnosed during adolescence were more likely to have completed fertility testing, suggesting that providers may be more likely to initiate fertility-related conversations with adolescent patients due to greater availability of cryopreservation options for older patients. Further, and unlike child-aged patients, adolescents are more able to engage in discussions surrounding fertility at the time of their cancer diagnosis, making them more attuned to their risks for infertility and subsequent efforts to test their fertility following treatment. It is also possible that survivors diagnosed during adolescence require more frequent oncology clinical visits during young adulthood to monitor for relapse, creating increased opportunities to initiate fertility-related discussions. Psychoeducation is warranted for all (long-term) survivors, regardless of age at diagnosis, to inform them of their infertility risk, fertility testing options, family building options, and to discuss alternatives to biological parenthood or a life without children. These conversations should be ongoing and tailored to survivors’ goals over time.

### Limitations

Findings from this study should be considered in the context of its limitations. Data were collected through self-report, and outcomes of fertility testing and risk for infertility may be inaccurate. The sample may not be generalizable to young adult survivors of childhood cancer outside of Germany, as systemic differences in healthcare delivery, access to contraception, and costs vary across countries. Additionally, there may be a selection bias, such that survivors with higher education completed surveys more frequently. No validated measure exists to measure uncertainty about fertility in cancer survivors, but the self-developed 3-item measure included in this study showed good internal consistency. Other single-items included in the survey were deemed to have sufficient face validity by experts in the field and were comparable to questions used to assess contraceptive behaviors in national studies. As is the case in all cross-sectional studies, the temporal relationship between some variables could not be tested. Finally, additional psychological variables (e.g., distress, development) were not assessed, and warrant further investigation to determine more factors related to the completion or interest in fertility testing.

## Conclusions

Young adult survivors of childhood cancer can have unique reproductive healthcare needs, including contraception to prevent unintended pregnancies and STIs, while also considering fertility testing to assess options for future biological parenthood. In our sample, the vast majority of survivors reported use of either single or dual methods of contraception. However, there is a large gap in care regarding fertility testing, with over half of the sample reporting interest, but < 15% having completed testing. As many participants were unaware of fertility testing options or wanted to wait until they were older, healthcare providers may need to discuss fertility testing multiple times after treatment. Further research in this area is needed to identify and address barriers to fertility testing among interested cancer survivors. Additionally, uncertainty regarding fertility was associated with both completion of, and interest in, fertility testing, suggesting a need for psychological support before and after fertility testing.

## Data Availability

The data that support the findings of this study are available from the corresponding author upon reasonable request.
